# Natural dietary compound naringin inhibits glioblastoma cancer neoangiogenesis

**DOI:** 10.1186/s40360-020-00426-1

**Published:** 2020-06-23

**Authors:** Sonia Aroui, Hamadi Fetoui, Abderraouf Kenani

**Affiliations:** 1 Laboratory of Biochemistry, Research Unit: UR 12ES08 “Cell Signaling and Pathologies”, Faculté de Médecine de Monastir, University of Monastir, 5019 Monastir, Tunisia; 2 Laboratory of Animal Eco-physiology, Faculty of Sciences, Sfax University, Sfax, Tunisia

**Keywords:** Angiogenesis, Naringin, Glioblastoma, Xenografts

## Abstract

**Background:**

Flavonoids, which existed nearly in all fruits and vegetables, are considered as a class of plant-secondary metabolites with a polyphenolic structure and have properties with health-improving potential. Yet, not so many experimental focus on the benefits of flavonoid in vivo after external application. Here we assessed the impacts of naringin in vitro and in vivo in the human glioma U-87 cells implanted into athymic mice.

**Methods:**

Tumor size and animal survival time were followed in naringin-treated mice bearing subcutaneous gliomas. To define the effects of naringin on angiogenesis, in vitro, tube formation and migration were assayed using endothelial HUVEC cell line.

**Results:**

Low concentration of naringin remarkably inhibited tubulogenesis and reduced cell invasion. Moreover, naringin has been shown to have a toxicity effect on U-87 cells in a dose-dependent way. Furthermore, naringin administration (120 mg/kg/day) applies serious anti-cancer belongings on glioblastoma, as demonstrated by a slow cancer progression.

**Conclusions:**

Our study has provided the first evidence on the antitumor effect of naringin, which is somehow due to the inhibition of invasion and angiogenesis.

## Background

For the past decade, the inefficiency of cytotoxic agents and drugs remains a paramount impediment for the successful treatment of human cancers. Glioma, the primary intracranial neoplasms, is one among such devastating cancers with 12–15 months of median survival rate [1]. Although the multi-modality treatments progress, including surgery, irradiation, and chemotherapy, glioblastoma multiforme (GBM) has retained their poor prognosis. However, the higher potency of tumor cells-infiltration limits treatment success because of its lead to the development of cross-resistance and treatment decline [2]. This necessitates further supporting chemotherapy for the cure of GBM.

Earlier, increasing attention has been given to the natural plant-compounds that reveal anti-tumor activity for human cancer. Among them, naringin (4′, 5, 7-trihydroxy flavanone-7-rhamnoglucoside), a glycone form of naringenin found in most citrus fruits [3]. This flavonoid has been reported to own numerous biological effects important to human health and reduce cardiac hypertrophy by inhibiting oxidative stress and inactivating c-Jun nuclear kinase (JNK-1) protein in type I diabetes [4]. It also ameliorates sodium arsenite-induced renal and hepatic toxicity in rats by modulating the activities of KIM-1, caspase-3, TGF-β and TNF-α [5]. Naringin inhibits the development of human breast cancer cells by targeting the β-catenin signaling pathway [6]. It also possesses the anti-apoptotic activity of hepatocellular carcinoma HepG2 cells [7] and prostate DU145 cancer cell line [8]. So far, our team has reported that naringin suppresses cell metastasis and modulates the level of matrix metalloproteinases (MMPs) via the downregulation of the ERK-P38-JNK signaling pathway in human glioblastoma cells [9]. Nonetheless, the anti-angiogenic activity of this flavonoid has not been well described. Attempts to new development, angiogenesis remain efficient pathways for the prevention and treatment of cancer [10]. Glioblastoma is the most vascularized malignant brain tumour since the survival of this tumor is depending on adequate blood provides [11]. Indeed, the development of a new tube network is associated with its degree of malignancy and treatment failure [12].

Herein, an in-vivo xenograft model and an in-vitro cell proliferation assay were used to evaluate the anti-tumor activity of naringin against GBM. The impact of naringin on the crown of xenografted glioma previously and subsequently to the inauguration of cancer was also assessed. These findings have led to exploring the efficiency of naringin as a promising therapeutic agent in glioma.

## Methods

### Chemicals

Naringin (98% purified) and all other reagents were purchased from Sigma Aldrich. All other chemicals were of analytical grade. Type I collagen was prepared as previously described [9]. Antibodies for VEGFR, p-VEGFR, AKT, p-AKT, ERK and p-ERK were purchased from Cell Signaling Technology (Danvers, MA, USA). Antibodies for GAPDH and the secondary antibodies were purchased from Santa Cruz Biotechnology (Santa Cruz, CA, USA).

### Cell culture

The human malignant glioma cell line, U87, was purchased Sigma-Aldrich (European Collection of Authenticated Cell Culture, ECACC, cat. no: 89081402, STR-PCR Data: Amelogenin:X; CSF1PO:10,11; D13S317: 8,11D16S539:12; D5S818:11,12; D7S820:8,9; THO1:9.3TPOX:8; vWA: 15,17.

Cells were maintained as a monolayer culture in T25 flask at 37 °C under the humidified condition with 5% CO2 and 95% air. The medium used for culturing the cells was DMEM medium with fetal bovine serum (FBS 10%) as a supplement, penicillin-G (50 units/ml), and streptomycin (50 μg/ml) were the antibiotics used.

Human umbilical vein endothelial cells (HUVECs) were isolated from fresh cords and cultured, as described by [13].

### Ethical approval

This study has obtained the approval of the Animal Ethics Committee (Comité d’éthique en experimentation animale COMETHEA accredited by the French legislation and European Union Directive (2010/63/UE).

### Xenograft mouse model

Athymic mice (Crl: CD-1 nuBR) (5 weeks old) were purchased from Charles River Laboratories (Oncins - SG, France). The mice were divided onto a group of chemopreventive effects (12 mice) and a group of therapeutic effects (12 mice). For the tumor establishment, the mice were injected on s.c into the right flank by 10^7^ U-87 cells suspended in serum-free DMEM (1% cellulose).

After seven days of tumor implantation, athymic mice were daily conducted by intraperitoneal administration (*i.p*) with naringin (60, 120, 180 mg/kg) or with saline (CMC-Na 5%, v/v) to determine dose-response screening. In order to assess the chemopreventiveeimpact of naringin, we treated animals by naringin, (120 mg/kg) 7 days before cell administration. However, the same dose was carried to mice subsequently to xenografts on the third day to assess the remedial effect of naringin.

Injections were performed daily up to the animals were sacrificed. Naringin was dissolved in 5% sodium carboxymethyl cellulose (CMC-Na) (sigma Chemical Co) and freshly prepared every day. Animals were survived daily for any health problem. The detectable tumor was measured every two days by a caliper. Tumor volume was determined by the equation: (height x length x width) continuously to the end of the manipulation.

### Detection of tumor suppression in vivo

Detection of tumor suppression in vivo was evaluated as described previously by [14]). The tumor inhibition ratio was calculated using the formula: T/C% = (mean tumor volume of the naringin-treated group on day Y/mean tumor volume of the control group on day Y) × 100 where T and C represent the ratio of the mean tumor volume in the treatment group and the mean tumor volume in the control group, respectively. The minimal T/Crrate represents the greatest tumor hindrance carry out.

### Cell viability assay

The U-87 and HUVEC cell viability were measured by the MTT assay. Briefly, cells were seeded onto 96-well plates overnight and, therefore, handled by different concentrations of naringin, diluted in culture media for an additional 48 h at 37 °C. Thus 100 ml of MTT (5 mg/ml stock in PBS) was added to each well and cells were incubated at 37 °C for another 4 h. Viable cells convert the soluble yellow MTT to insoluble purple formazan by the action of mitochondrial succinate dehydrogenase. 100 μl of solubilizing solution (acid isopropanol) was added, mixed well and the color developed was read at 650 nm in an ELISA reader. The percentage of viability of cells was calculated and plotted on a graph. From the assay, the IC50 value for naringin was observed. The IC50 represents the dosage of the drug at which inhibition of 50% cell growth.

### Migration assay

In the Transwell migration assay, the HUVEC cells treated with naringin for 24 h were seeded on the Collagen type I coated at lower chambers of 8 mm pore size polycarbonate membrane (Neuro Probe, Cabin John, MD, USA) forr2 haatt37°. Cellss were then trypsinated from culture flasks and suspended in F12 with 5% fetal bovine seruma at 5x10^5^cells/ml. Aliquots of 2200 ml of the HUVEC cell suspension, containing either naringin or vehicle alone, were added to the upper chamber.

The cells that invaded to the lower surface were fixed for 15 min with 4% paraformaldehyde. Then rinsed in PBS thrice and the invaded cells were stained with 0.2% crystal violet for 10 min. Five images were captured separately for every treatment and the averages were quantified using Image J software.

### Neo-tubulogenesis assay

Tubulogenesis was performed using ccollagen as thee extracellular rmatrix on a 24-well plate. 300 ml of collageng gel was added for each well and kept at 37 °C for polymerization. The HUVEC cells were seeded then in the collagen-well at a number of 5 × 10^4^ cells/well and cultured again att37°C for 18 h. After that, we removed the culture medium and 300 ml of collagen gel were drained upper of the first collagen gel and kept for polymerization for again 10 min att37°C. A new medium with naringin at the appropriate concentration (or saline) was added. The action was repeated every day. The cells were incubated for 4 days at 37 °C. We photographed each well and then we quatified the tubulogenesis by measuring the tubular length of the cells in six different areas using the Image J software.

### Western blotting assay

Total proteins were extracted using radio immunoprecipitation assay (RIPA) buffer (Cell Signaling technology, Danvers, MA, USA) supplemented with phosphatase inhibitor and protease inhibitor (Selleck Chemicals, Houston, TX, USA). Then proteins (50 μg/lane) were separated by 10% SDS-PAGE gel and transferred to PVDF membranes. The membranes were blocked with 5% dried milk in TBST buffer and incubated with the corresponding primary antibodies at 4 °C overnight. Next, the membranes were incubated with secondary antibodies for 2 h and the bands on the membranes were visualized using an enhanced chemiluminescence (ECL) reagent (GE Healthcare, Hatfield, UK).

### Neo-angiogenesis assay in vivo

We measure the concentration of hemoglobin in implanted tumors as described by [15] in order to quantities neo-angiogenesis.

### Haematoxylin and eosin (H&E) staining and immunohistochemistry (IHC)

After being collected, tumor tissues were attached in formalin buffer (10%) for 24 h, fixed in the paraffin and then fragmented for IHC studies. The obtained sections were then cut and deparaffined. All the sections were fixed for 10 mn with antigen and then incubated overnight with an anti- CD31 antibody (ab28364; Abcam, Cambridge, MA, USA) at a concentration of 1:100 at 4 °C. Before incubation with a secondary antibody for 30 min, sections were washed by a Tween buffer for 2 h. By the end, the nuclei were stained by hematoxylin. Six fields of microvascular were haphazardly selected from ech slide and visualized at 200× magnification.

### Isolation of RNA for qRT PCR

5 X 104 cells were used for total RNA extraction using TRIZOL reagent. qRT-PCR was carried out to measure the mRNA levels of CD105, CD31, and β-actin with real-time PCR system (Model no: CFX96, Bio-Rad Laboratories) using SYBR Green master mix. The sequence for, CD105 is Forward 5′ AGAAGGCTGTGTTCTTCGCA 3′, Reverse 5′ AAAGGCAGCGTCTACTTGCT 3′, for CD31 is Forward 5′ GATCCCCAGAGCGTTACTCG 3′ Reverse 5′ GTTGTGGAAACTCACACGCC 3′ and for β-actin is Forward 5′ CTCTGTGTGGATTGGTGGCT 3‘ and backward 5’ CGCAGCTCAGTAACAGTCCG 3′. The cycle threshold (Ct) values were determined using qbase PLUS software. The ΔCT value of relative quantification was carried out to find the fold changes in expression (ΔCT = ΔCT reference – ΔCT target).

### Euthanasia and organs collection

Athymic mice in each treatment condition was euthanized by overdosing them with CO2. Immediately after euthanasia, the organs were harvested and rinsed with PBS. IVIS imaging system was harvested for neoangiogenesis imaging assay. After the image acquisition, the tissues were transferred to a vial containing 1 ml PBS and frozen until tissue homogenization or embedded in a plastic cassette containing optimal cutting temperature medium and slowly cooled over dry ice and paraffin for IHC assay.

### Statistical analysis

All the data were processed with SPSS 16 software and Graph Pad Prism 5, USA, software.

The results were expressed as mean ± SD. One way ANOVA followed by Tukeys’s post-hoc.

Test. *P* < 0.05 was considered statistically significant.

## Results

### In vivo anti-tumor efficacy of naringin

**Fig. 1:**
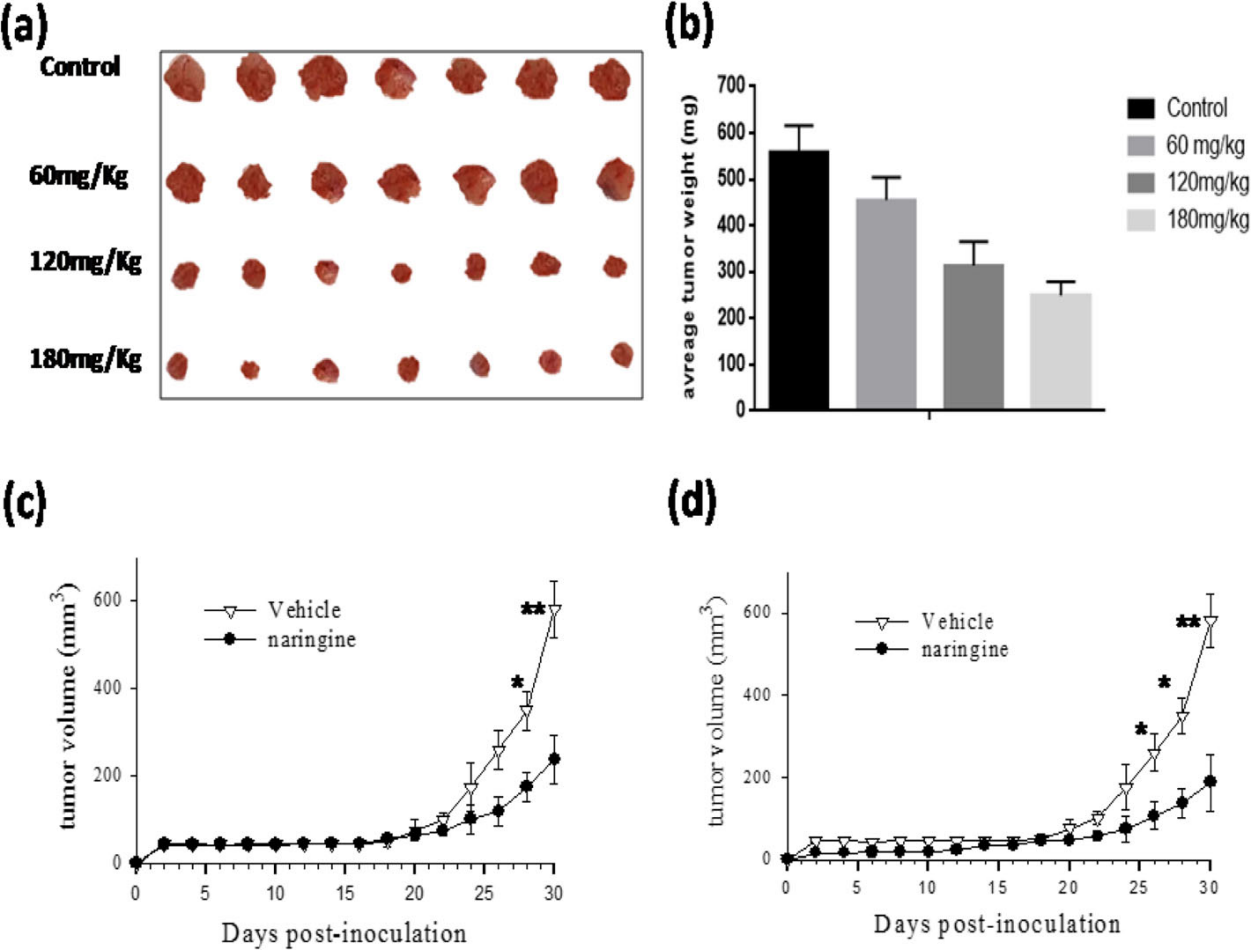
Images of tumors harvested from the mice after the 20-day experimental period (**a**); average tumor weight of the mice after 20 days (**b**). Naringin (60, 120 or 180 mg/kg) administrationnbegan 7 days-after U-87 inoculation-into the right-flank of the nude athymic mice, and theinjection was repeated until-the animals were-sacrificed. Chemopreventive (**c**) and-therapeutic effects (**d**) of naringin on-human-glioma tumor xenografts-grown s.c. naringin (120 mg/ kg/day). The administration began 7-days-before the U-87iinoculation (chemopreventiveeeffect) or 3 days after cell implantation (therapeutic effect). The tumor size was determined twice a week. Daily8average tumor8volumes for each group were8compared throughout the experiment usinggStudent’s *t*-test. *p < 0.05, **p < 0.01

**Fig. 2:**
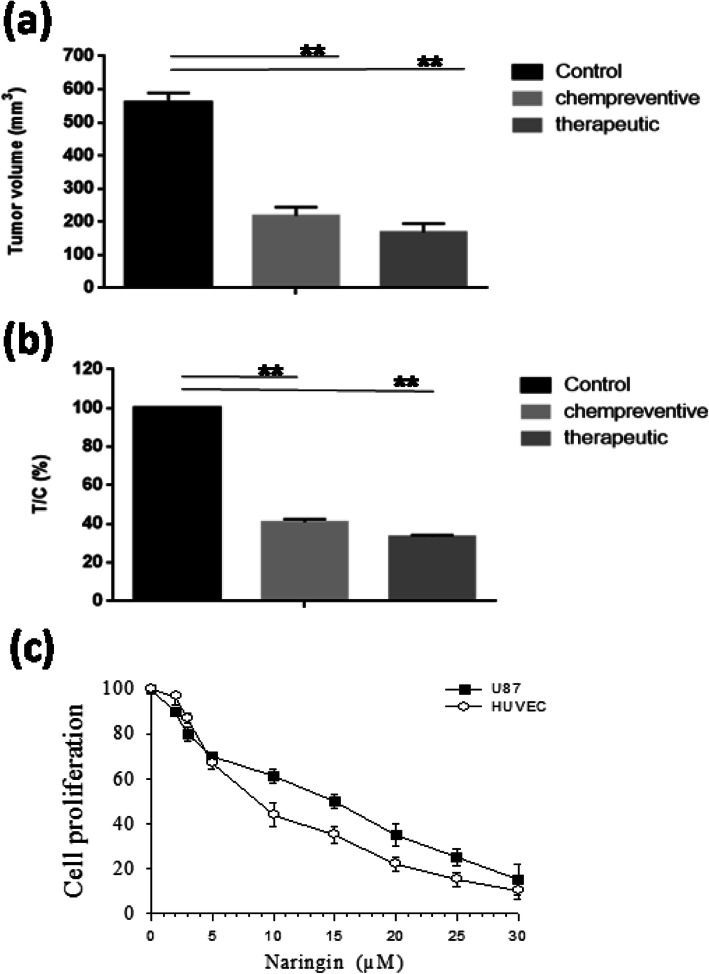
In-vivo*-*anti-tumor activity of naringin given i.p. against s.c. Implantedhhumann malignant glioma. Naringin (120 mg/kg/day)8administration began 7 dayssbefore U-87 inoculation (chemopreventive effect) (**a**) or 3 daysaafter celliimplantation (therapeutic effect) into theeright flank of the nude athymic mice (**b**) and8the6injection was done again until the day of sacrifice. Dose-6dependent6inhibition of human6glioma cellss(U-87) and HUVEC proliferation by naringin was measured by MTT assay (**c**). Resultssrepresent the means of five mice inneach group anddare expressed as averageevolume (mm^3^) ± SEM. Daily average tumorrvolumes forreach group were6compared throughout6the experiment usingsStudent’s *t*-test. **p < 0.01

Glioblastoma was chosen as a model of cancer to investigate the anti-tumor efficacy of naringin. The antitumor growth effect in vivo was evaluated firstly by measuring tumor volume following treatment with the different doses of compounds (60, 120 and 180 mg/kg). The analysis was seated on day 3, subsequently to xenograft, as shown in the method section. Data demonstrated that therapy with 120 mg of naringin was efficient than small concentrations, as exhibited by rapid tumor installation rate. Although, we have not detected a significant difference in tumor gain between the 120 and 180 mg/kg/day (Fig. 1a and b). Therefore, for the next experiments, 120 mg/kg/day of naringin have been used as an adequate dose. Secondly, we assessed both therapeutic and chemopreventive-effects of naringin on U87 implanted cells. For the chemopreventive-response of naringin, we began the analysis 7 days previously to the inculcation of tumor cells. In comparison, therapy with naringin was initiated 3 days after tumor cell injection to assay the therapeutic effect. In the chemopreventive group, mice that received naringin possessed remarkably smaller tumors as compared to control mice. Such a difference was observed from the beginning point until the term of the experiment (Fig. 1c). Besides, in the therapeutic group, mice that treated with naringin also displayed remarkably lower tumor volume than the control mice since the 25th day until the term of the experiment (Fig. 1d). By the end of the experiment, we have noticed that the ratio T/C-values which were used to determine tumor response, were similar between naringin-treated mice in the chemopreventive8-group and the therapeutic group (40.8 and 33.2%, respectively). The results given in Fig. 2a and b illustrate that naringin was capable of performing efficient gliomas- suppress sooner or later to tumor founding.

### Effects of naringin on the viability of U-87 glioma cells and HUVEC endothelial cells

To effect of naringin on cell viability of human glioma xenografts was studied by MTT assay at various concentrations (Fig. 2c). Naringin inhibited 50% of cell proliferation (IC50) at 15.1 ± 1 and 10.8 ± .2 μM for_U-87 and HUVEC cells, respectively. Our finding suggest that HUVEC cells are slightly but significantly more sensitive to naringin as compared withhU-87 cells (Student’s t-test, *p* < 0.05). Our results showed that naringin could have an anti-angiogenic effect.

### Matrigel invasion assay

**Fig. 3:**
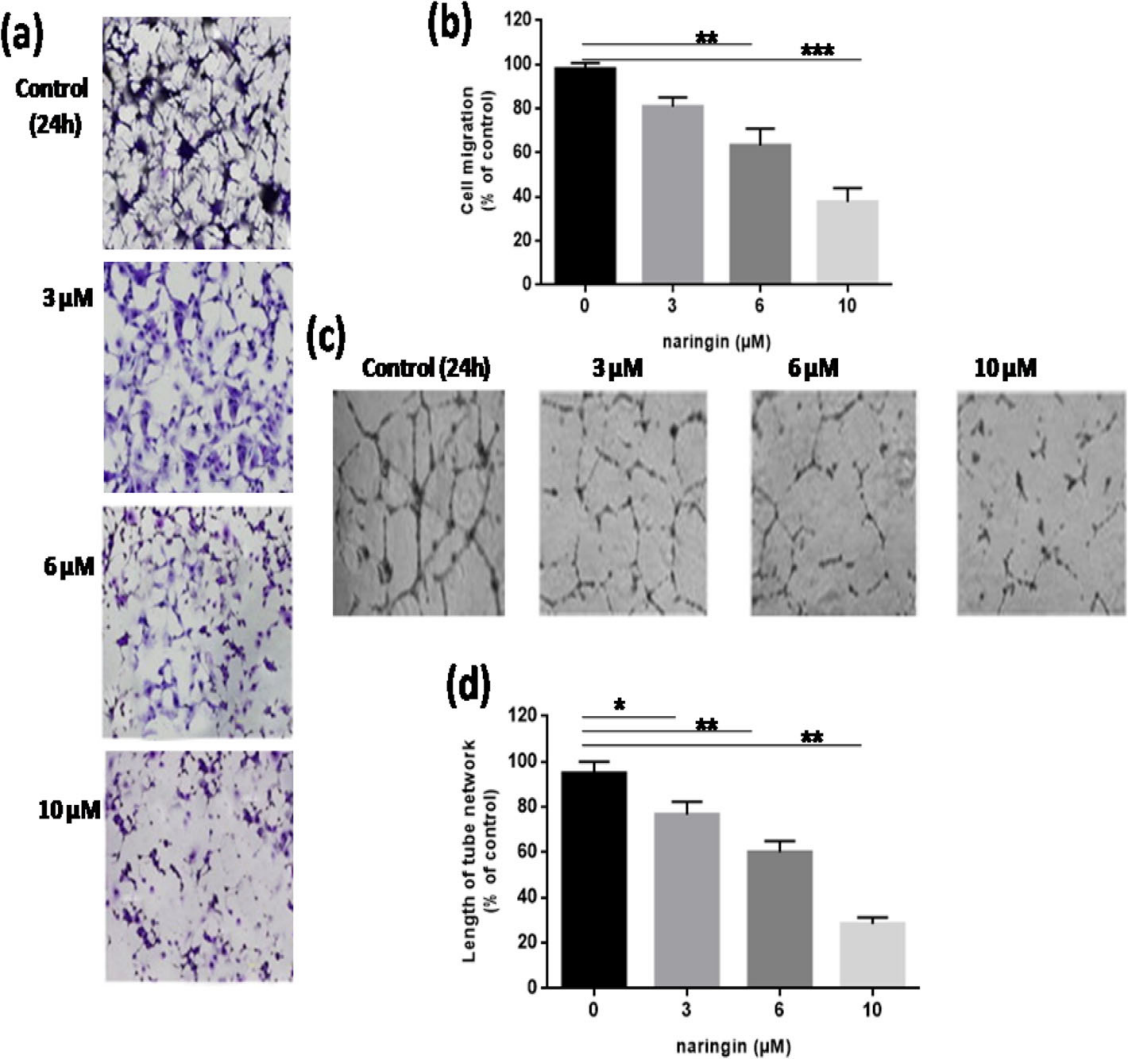
Matrigel transwell invasion assays were used to measure cell invasiveness (**a b**). Following incubation with Naringin (0, 3, 6, and 10 μM) for 24 h, cells that invaded through the membrane were stained and representative fields were photographed.) Tube formation assay; the ability of flavonoid to6inhibit vessel blood structureeformation on6collagen was assessed as5described in Material and methods (**c**, **d**) Data-_are presented6as themmean6tube6length *per6*field of microscope compared with that observed with untreated6cells. Bar graphs represent the mean number of invaded cells from ten random fields, and all results are representative of three independent experiments (mean ± SD). *P value< 0.05 and **P value< 0.01 and ***P < 0.001 vs. control group (untreated cells).

In the Transwell invasion assay, the HUVEC cells treated with naringin for 24 h were seeded on the matrigel (Millipore) coated top chambers. In the lower chambers, serum-containing DMEM medium was added and the FBS in the medium acts as a chemo-attractant. After incubation for 48 h, the non-invading cells in the top chamber were removed with a cotton swab. The cells that invaded to the lower surface were fixed for 15 min with 4% paraformaldehyde. Then rinsed in PBS thrice and the invaded cells were stained with 0.2% crystal violet for 10 min. For all the groups, more than five image fields were photographed and the average was quantified. HUVEC invasion was blocked in a dose-dependent manner by naringin with an IC50 of 5.3 μM (Fig. 3a and b).

### Downregulation of neovascularization by naringin

Morphological differentiation of endothelial cells after naringin treatment was explored through the tube endothelial neo-formation assay. HUVEC were tested to generate tube-cords in a collagen gel. The naringin-efficiency effect was estimated by checking the length of the tube complex generated. As demonstrated in Fig. 3c and d, Naringin treatment decreases the diameter and wideness of endothelial8tubular structures, respectively, in a dose-dependent manner with an IC50 of 11.2 μM.

### Naringin suppressed VEGF-induced activation of VEGFR2 and the downstream molecules

**Fig. 4:**
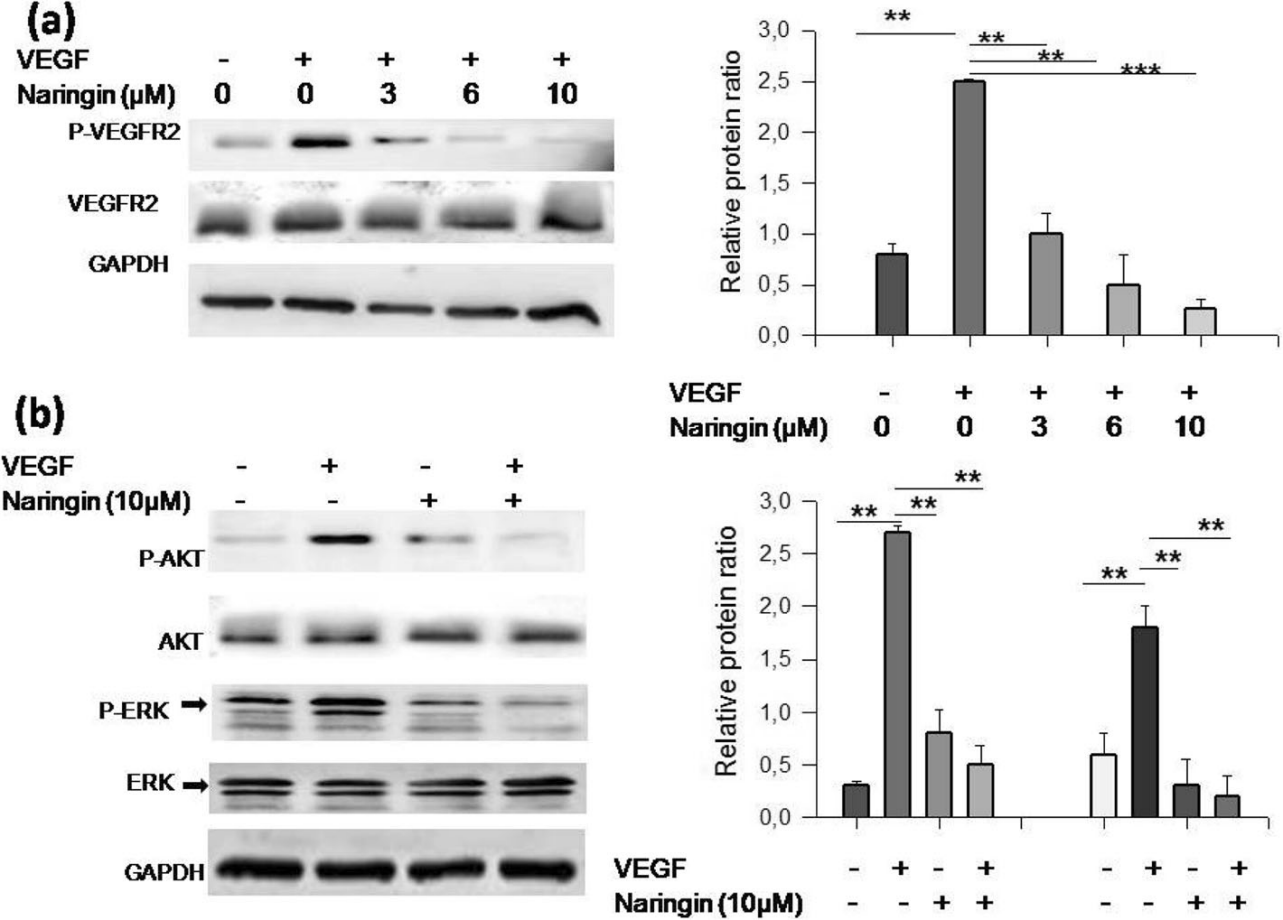
Naringin inhibits VEGFR2 signaling. HUVEC cells were incubated in the presence or absence of naringin followed by stimulation with VEGF for another 5 min. Phosphorylation of VEGFR2 (**a**), AKT and ERK (**b**) was assessed by western blot. GAPDH level was used as a loading control. Results are representative of two to four experiments (mean ± SD). **P value< 0.01

Vascular endothelial growth factor (VEGF) is one of the most important pro-angiogenic factors which act via stimulation of VEGFR2, the main tyrosine kinase receptors on the endothelial cell surface [16]. Activation of the VEGFR signaling pathway is dependent on its binding with its VEGF ligand. Accordingly, we examined in this study whether Naringin could inhibit the activation of VEGFR2 after VEGF stimulation. HUVEC cells were pretreated with Naringin for 6 h and then stimulated with VEGF for 5 min. The results obtained showed that VEGF stimulation increased the protein level of p-VEGFR2, however naringin inhibits the phosphorylation of VEGFR2 induced by VEGF in a dose-dependent manner (Fig. 4a). Subsequently, VEGF stimulation up-regulated the level of p-AKT and p-ERK, whereas naringin greatly diminish VEGF-induced phosphorylation of AKT and ERK (Fig. 4b). Altogether, our results showed that naringin could inhibit ligand-induced activation of VEGFR2 and phosphorylation of downstream signals AKT and ERK.

### Downregulation of glioma-induced angiogenesis in vivo by naringin

**Fig. 5:**
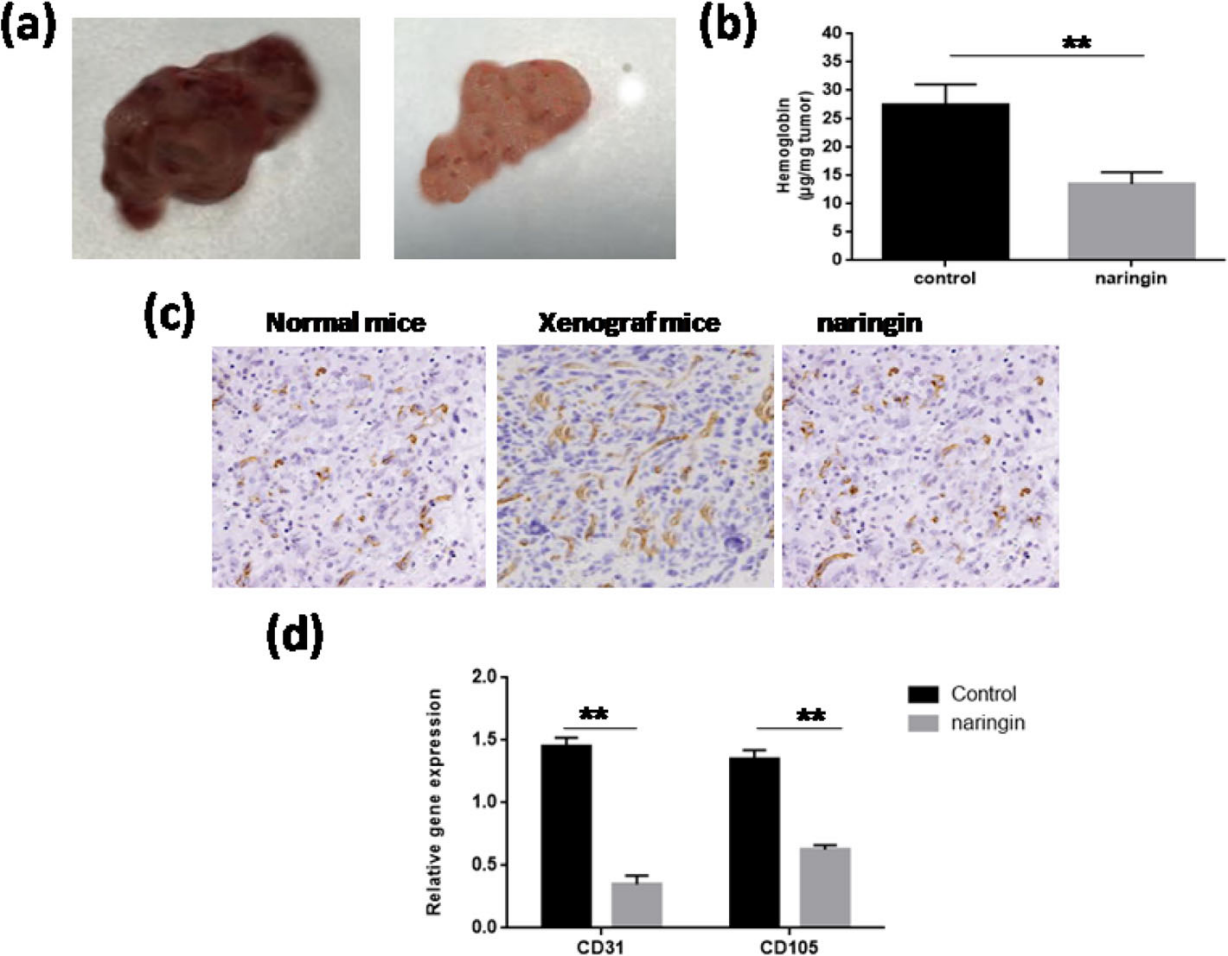
In vivo downregulation of glioma-induced angiogenesis; angiogenesis assay. S.c. tumors were implanted in athymic nude mice and were removed and photographed after30 days. Application of naringin (120 mg/kg/day) or with saline alone was done every day until the6animals were6sacrificed. **a** Quantification of new vessel development by assessment of hemoglobin in cancers as mentioned in Material and methods. **b** An illustration of s.c.gliomas was presented. **c** IHC staining of CD31 shows that naringin prevented glioblastoma cancer angiogenesis. **d** MRNA6expression of6CD105 and CD31 in s.c. tumors. ANOVA was performed using the mean fold change compared with control. **P value< 0.01

To determine the efficiency of naringin to inhibit the microvasculature formation in vivo, hemoglobin-level and IHC staining of CD31 were checked in s.c. Tumors. After 30 days of xenografts initiation in athymic nude mice, tumors were removed and captured. In non-treated mice, tumors appeared red. Nonetheless, in naringin-conducted mice, tumors were pink, expressing lower neo-vasculature formation in comparison with the non-treated mice (Fig. 5a). Hemoglobin quantity was also measured to estimate new vessel blood formation of both tumors. By comparison with the control group, naringin accordingly repressed the hemoglobin quantity to 18.2 μg/mg (Fig. 5b). The microvasculature was detected by IHC staining of CD31 and was shown denser in the non-treated group than in the control group (12.470 ± 1.332 vs. 7.400 ± 1.908, *p* = 0.020) (Fig. 5c). However, treatment by naringin significantly reduced the density of the microvasculature compared to the non-treated group (5.700 ± 3.535 vs. 12.470 ± 1.332, *p* = 0.024). Also, the mRNA expression of CD311 and CD105 was significantly decreased. CD311 was decreased by 88% compared with 65% for CD105, in naringin-treaded s.c. tumors (Fig. 5d). Altogether, our results demonstrate that naringin is apt to prevent glioma-induced angiogenesis in vivo.

## Discussion

Naringin is a flavonoid found in citrus fruit with various biological and pharmacological activities, including antioxidant and anti-cancer related properties. The precise molecular mechanism of anti-invasive and angiogenesis effects of naringin has not been identified yet. It is well known that there are no previous reports about the anti-invasive and anti-angiogenic ability of naringin against GBM. In the current study, we have characterized the chemopreventive and the therapeutic effect of Naringin on the glioblastoma xenografts*.*

For the purpose, that angiogenesis has been mediated to make a crucial part in glioblastoma and since it requires a network proliferation of endothelial cells from parent blood vessels [11, 12], we promote the study of the effect of naringin on the propagation of umbilical blood vessel endothelial cells. Our results proved that naringin repressed the development of the endothelial HUVEC cells in a concentration-dependent manner with a lesser IC50ccompared with that raised on U87 glioma cells. Such IC50 was already reported for naringin with other cancers such as osteosarcoma [17], prostate cancer [18], intestinal cancer [19] and glioma [20] suggesting that extended surgery with naringin could be possibly advantageous strategy for gliomas prevention and treatment.

Thus, we farther explored the anti-angiogenic effects of this natural product. Our issues have shown that naringin could inhibit in vitro neo-angiogenesis. Many researchers have demonstrated that this process is a pivotal occurrence for the offshoot of cancer cells and their reaching into othert tissues, a phenomenon called metastasis.

Interestingly, in vitro attempts found that the inhibitory impact of naringin on these trials is owing to the down-regulation of both basic occurrences hidden angiogenesis, (i) endothelial-cell invasion-across the ECM and (ii) morphogenic-differentiation of HUVEC cells into blood vessels-like building.

In this study, we have shown that naringin exerted significant antitumor effects on s.c.gliomas at a dose of 120 mg/kg/day. Previous works have delineated that i.p. administration of 115 mg/kg of naringin to mice achieved a peak plasma level of 15 μM after 20 min, which declined within 2 h [21]. Comparable plasma-level is corresponding to the doses used in our experiments to repress neo-angiogenesis in vitro. In addition, toxicity and pharmacokinetics-effects of naringin were expressed in human and great-oral naringin intake (621 μM) appears without treatment-related toxicity. Besides, the serum concentrations of naringin usually peaked at 12 min and always denied within 1 h. The balance peak serum dose, subsequently administration of naringin was 20 μM [22].

In the HUVEC cells, naringin mediated regression of tubulogenesis and cell invasion line was shown at doses (between 5 and 11 μM) that may be created over dietary intake of the natural product. Such inhibition of formation of new vessels branches is a consequence of reduced expression of many angiogenic factors like VEGF in endothelial vascular cells which is congruent with the our finding [23]. Various researches reported the pivotal role of VEGFR2 in tumor angiogenesis and metastasis and the ability of flavonoids to disturb VEGF-related cell signaling in cancer cells [24].

Stimulation of VEGFR2 induce the phosphorylation of different downstream signals, as in as p38 mitogenic activated protein kinases (p38MAPK), phosphoinositide 3-kinase (PI3K),, extracellular signaling regulated kinases (ERK 1/2) and protein kinase B (AKT), proceeded by activation of endothelial cells [25]. Thus, the VEGF and VEGFR signaling pathways are handsome purpose for anticancer therapeutics.

Moreover, naringin was demonstrated to down-regulate CD31aand CD105 mRNA, two-endothelial cell markers from recently produced blood vessels. Hemoglobin exciting in glioma tumors was also decreased. Altogether, these results firmly reinforce the approach that, in vivo, naringin could be an angiogenesis determent.

Herein, we have also proved that naringin can reduce the progress of s.c. Gliomas in athymic-mice previously and back of the establishment of tumors as shown by moderate tumor progress ratio. Moreover, naringin enhances tumor growth suppression and mice endurance time in mice carrying glioblastoma. Other researchers in vivo had also considered the preventive impact of naringin. Yu and all showed that naringin prevented intestinal tumorigenesis, breast cancer, prostate cancer and melanoma [26].

As well, there are minor studies that have illustrated the anti-tumoral potential of naringin on tumor gain in vivo when carrying out as a therapeutic agent [27].

## Conclusion

This study offers a new understanding of how naringin could be advantageous after the lesion has been established.

In view of the high vascularization of malignant gliomas and since the growth and survival of these tumors are dependent on a suitable blood vessels supply [28], our results demonstrate the considerable amount of interest owing to naringin and its relevance to either therapeutic or chemopreventive employment in brain cancer.

## Data Availability

All the data and materials are available and updated.

## Abbreviations

GBM: Glioblastoma multiforme
H&E: Haematoxylin and eosin staining
IHC: Immunohistochemistry
